# Polycystic ovary syndrome induced by exposure to testosterone propionate and effects of sympathectomy on the persistence of the syndrome

**DOI:** 10.1186/s12958-017-0267-0

**Published:** 2017-07-10

**Authors:** Leticia Morales-Ledesma, Juan Antonio Díaz Ramos, Angélica Trujillo Hernández

**Affiliations:** 1 Biology of Reproduction Research Unit, Physiology of Reproduction Laboratory, Facultad de Estudios Superiores Zaragoza, UNAM, AP 9-020, CP 15000 México, D. F, México; 2 Benemérita Universidad Autónoma de Puebla, Facultad de Ciencias Biológicas, Edificio BIO1, Ciudad Universitaria, Boulevard Valsequillo y Avenida San Claudio S/N. C.P, 72570 Puebla, Puebla México

**Keywords:** Polycystic ovary syndrome, Testosterone propionate, Superior ovarian nerve

## Abstract

**Background:**

Polycystic ovary syndrome is a complex disorder affecting 6 to 10% of women of reproductive age. In recent years, the cause of the syndrome has been associated with increased androgen serum levels, as well as sympathetic nervous system hyperactivity. The present study analyzed the effects at birth of a single testosterone propionate dose in rats, as well as the role of the superior ovarian nerve in polycystic ovary syndrome persistence.

**Methods:**

Newborn female rats of the CIIZV strain were injected with a single dose of 100 μg testosterone propionate, or corn oil as a vehicle. At 24 days of age, rats were subjected to superior ovarian nerve unilateral or bilateral section. A group of animals was sacrificed on their first vaginal estrus, and a second group was sacrificed at 90 days of age, when they presented an estrus preceded by a proestrus.

**Results:**

Testosterone propionate administration at birth blocked ovulation both in adult animals and in animals sacrificed on the first vaginal estrus. Histological analysis of testosterone propionate-treated adult rat ovaries revealed the development of follicular cysts. Ovulation could not be restored by unilateral or bilateral section, and ovaries presented follicular cyst and absence of corpora lutea.

**Conclusions:**

Our results suggest that testosterone propionate treatment at birth results in the development of polycystic ovary syndrome, and that this outcome is not due to superior ovarian nerve noradrenergic innervation hyperactivity.

## Background

The administration of androgens (testosterone, testosterone propionate, androstenedione) or estrogens (estradiol valerate, EV) is a useful tool to generate animal models developing a type of physiopathology similar to that observed in women with polycystic ovary syndrome (PCOS) [1–7]. PCOS is the most common and least understood endocrine disorder affecting approximately 5% of women of reproductive age [8], and it consists of an endocrine disorder characterized by hyperandrogenism and anovulation; it has been associated to hypothalamus-hypophysis-ovary axis alterations, as well as other metabolic alterations [9–15]. Excess androgens (originated by the mother or the fetus) in early embryo development stages could explain the onset of PCOS in the adult animal [1, 16–21]. In rats and monkeys, prenatal or neonatal exposure to high androgen doses induces abnormal follicular growth during the animal’s adult life, resulting in cyst formation and, consequently, anovulation [16, 22, 23]. Androgens act on the androgenic receptors (AR), and use this path to perform important functions for follicular development and women fertility [24]. Such participation has been evidenced by in vivo studies using various animal models [25, 26]. In female mice, AR mutations result in premature ovarian damage induced by impaired folliculogenesis [25–27].

Recently, Caldwell et al. [28] have shown that global loss of the AR in knockout mice (ARKO), does not result in a PCOS induced by dihydritestosterone (DHT). In mice with neuron-specific loss of AR (NeuroARKO), the treatment with DHT resulted in acyclicity, but not in the development ovulatory dysfunction. However, the authors did not fully protect against the development of DHT-induced cystic follicles. These results demonstrate that extra ovarian neuroendocrine AR sites of androgen action are predominantly involved in the pathogenesis of PCOS.

At the beginning of the 1990’s, PCOS was proposed to be a response of increased activity in the sympathetic innervations of the ovary [3]. Histofluorescence analyses of rat ovaries with EV-induced PCOS revealed a high catecholaminergic innervation density, which was attributed to an increase of neural growth factor (NGF) in the ovary [2–4]. The decrease in sympathetic activity produced by superior ovarian nerve (SON) bilateral section results in a decrease in the concentration of noradrenaline, followed by the recovery of ovulation cycles and ovarian function [29]. Moreover, our research group has shown that SON unilateral section in animals with EV-induced PCOS restores ovulation in the innervated gonad, but not in the denervated one [30].

Because little is known about how androgen signaling contributes to pathophysiological ovarian conditions, this work aimed to evaluate the effects of exposure to testosterone propionate (TP) on female rats at birth on: 1) the steroid hormone secretion, 2) gonadotropin secretions, and 3) the presence of follicular cysts. Considering that it has been postulated that sympathetic ovarian innervation is a modulator of ovarian function [31–36], we also evaluated the role of the sympathetic innervation running through the SON in PCOS persistence at pubertal and adult rats.

## Methods

### Animals

We placed CII-ZV strain newborn rats under controlled light and dark (lights on from 05:00 to 19:00 h.) and temperature (22 ± 2 °C) conditions. All experiments were performed in strict accordance with the Mexican Law of Animal Treatment and Protection Guidelines and the specifications of the Mexican Official Standard.

NOM-062-ZOO-1999 for production, care, and use of laboratory animals.

### Assessment of estrus cycle

The animals’ estrus cycle was monitored, by examining vaginal lavage, after the presence of vaginal canalization, and for 2 weeks before the animals were euthanized. Following Marcondes et al.’s [37] methodology, every morning, between 9 and 10 am, vaginal smears were obtained with a sterile 3 mm inoculating loop. The inoculating loop was soaked in normal saline, placed on a standard slide, stained with hematoxylin-eosin, and observed under a light microscope.

### Testosterone propionate (TP) treatment

On their birth date (day zero), rats were distributed into groups of seven individuals (six females and one male; the male was removed when the animals were 30 days old.). Between 8:00 and 10:00 h, females received an only dose of 100 μg testosterone propionate (Sigma Chemical Co., St. Louis Mo. USA) by subcutaneous injection, dissolved in 0.05 ml commercial corn oil, which served as a vehicle (VEH). All rats were allowed free access to their mother until weaning (day 24), after that, they were allowed free access to water and food until the day of the autopsy.

### Superior ovarian nerve section

At 24 days of age, animals treated with TP or VEH were subjected to left (SONL), right (SONR), or bilateral (SONB) superior ovarian nerve sections. Animals were anesthetized with ether, and the incision area was cleaned with surgical soap and shaved. An approximately one-centimeter long skin and muscle dorsal incision was made, through which the uterus-attached ovary was extracted to locate the suspensory ligament, which runs along the superior ovarian nerve. The section was performed, ovary and uterus were returned to the peritoneal cavity, and the surgery was finished by layer suture.

### Autopsy

On the first vaginal estrus (FVE), or on the 90th day of age (vaginal estrus preceded by proestrus), animals were subjected to autopsy by decapitation between 8:00 and 10:00 h. We verified that ovaries in animals subjected to unilateral or bilateral SON section were free in the abdominal cavity. Oviducts were dissected, and ovocytes were detected and counted using a stereoscopic microscope, as per standard laboratory procedures.

Trunk blood was collected and kept at room temperature for 30 min, and then centrifuged at 3500 rpm for 15 min. Serum was separated from cell buttons and stored at −20 °C in Eppendorf tubes until progesterone (P4), 17β-estradiol (E2), follicle stimulating hormone (FSH), and luteinizing hormone (LH) quantifications were performed.

### Steroid hormone and gonadotropin quantification

E2 and P4 serum concentrations were quantified by solid phase RIA using commercial Coat-A-Count reagents (Diagnostic Products Corp., Los Angeles, CA, USA). The LH and FSH levels in the serum (ng/ml) were measured using the double antibody RIA technique, with reagents and protocols kindly supplied by the NIADDK National Pituitary Program (Bethesda, MD, USA).

### Ovarian morphology

Ovaries were fixed in Bouin’s solution, dehydrated, and included in paraffin. 10-μm thick sections were obtained and stained with hematoxylin-eosin. Follicular cyst presence was analyzed as a PCOS development indicator. The criteria for cysts were: follicles that presented a wide antral cavity, granulosa cell layer decrease, thecal hyperplasia, and absence of ovocyte [3, 4, 22].

### Statistical analysis

Hormone concentrations were analyzed with an ANOVA (multifactorial variance analysis) test followed by a Tukey’s test. A Student’s T test was used to compare two groups. The number of ova shed was analyzed using a Kruskal-Wallis test followed by a Mann-Whitney U test. The ovulation rate (the number of animals ovulating divided by the total number of animals times 100) was analyzed using a Fisher’s test. Differences in probabilities equal or less than 0.05 were considered statistically significant.

## Results

### Effects of testosterone propionate administration

The administration of TP at birth blocked the first ovulation in 100% of the animals. The same effect was verified when animals were sacrificed at 90 days of age.

The animals injected with TP showed high testosterone serum levels when they were 90 days old (VEH 29.0 ± 5.4 ng/ml vs TP 98.2 ± 9.1 ng/ml, *p* < 0.05 Student’s “t” test).

In their first vaginal estrus, VEH-treated animals showed a larger number of ova shed by the right ovary compared to ovocytes released by the left ovary (Right ovary: 10.6 ± 0.9 vs. Left ovary: 7.6 ± 0.8; *p* < 0.05; Kruskal-Wallis test followed by Mann-Whitney U test). The same effect was detected in adult age (Right ovary: 7.7 ± 0.5 vs Left ovary: 5.2 ± 0.7; *p* < 0.05; Kruskal-Wallis test followed by Mann-Whitney U test).

**Fig. 1:**
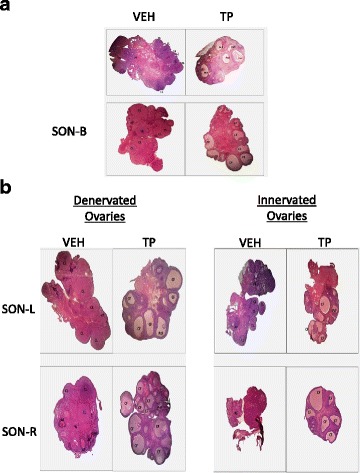
Adult rat ovaries (90 days of age). **a** Ovaries of animals treated at birth with corn oil vehicle (VEH) or testosterone propionate (TP). Lower panel shows adult rat ovaries of animals injected at birth with VEH or TP and subjected to bilateral SON section. **b** Denervated and Innervated adult rat ovaries; animals were injected at birth with VEH or TP and subjected to unilateral SON section. CL: Corpus luteum, PF: primordial follicles, PcF: precystic follicles, AF: atretic follicles, CF: cystic follicles

In adult animals (90 days of age), the treatment with VEH resulted in the presence of typical healthy ovary structures, with follicles at all developmental stages, and presence of corpora lutea. Contrastingly, the histological analysis of ovaries from TP-treated animals showed the development of follicular cysts, a lack of the follicular reserve, and absence of corpora lutea (Fig. 1a).

At 90 days of age, groups injected with TP showed irregular estrous cycles, characterized by vaginal estrus persistence, which was not observed in the VEH-treated group.

**Fig. 2:**
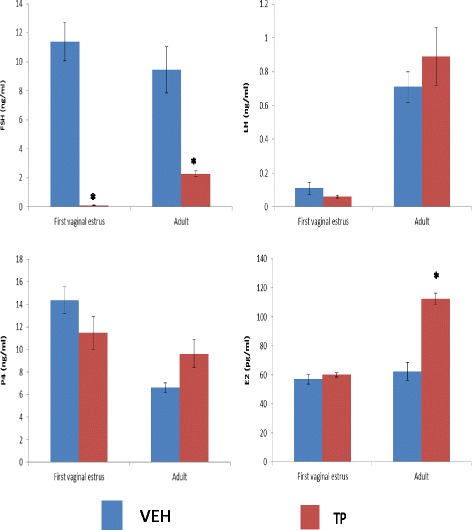
Mean ± s.e.m. of FSH, LH, progesterone (P4), and estradiol (E2) serum levels in females injected with vehicle (VEH) or testosterone propionate (TP) at birth, and sacrificed on the first vaginal estrus or in adult age. *p < 0.05 vs. VEH group. Student’s “t” test

The group of animals receiving TP at birth and sacrificed on the first vaginal estrus presented lower FSH serum levels compared to the VEH-treated group (Fig. 2). There were no changes in LH, P4, and E2 secretion (Fig. 2). When animals were sacrificed at adult age, FSH serum levels were lower in the TP-treated group than in the VEH-treated group. Serum levels of LH and P4 were not modified, whereas E2 levels increased compared to the VEH-treated group (Fig. 2).

### Effects of unilateral or bilateral section of the SON

As described above, TP treatment at birth results in blocked ovulation in pubertal and adult animals, and ovulation is not restored after unilateral or bilateral SON section.

At the first vaginal estrus, the left or right SON section resulted in a decrease in the number of ova shed by the denervated ovary compared to the VEH-treated group without SON section (Table 1). In adult age, only the left SON section resulted in a reduced number of ova shed by the denervated ovary, in comparison to the ovary of the same group with intact innervation (Table 1).

**Table 1:** Mean ± s.e.m. number of ova shed by the ovary in females injected with vehicle (VEH) at birth, and subjected to left superior ovarian nerve section (SONL), right ovarian nerve section (SONR), or bilateral nerve section (SONB) at 24 days of age, and sacrificed on the first vaginal estrus or in adult age

Groups	First vaginal estrus			Adult		
	n	Left ovary	Right ovary	n	Left ovary	Right ovary
VEH	10	7.6 ± 0.8	10.6 ± 0.9*	10	5.2 ± 0.7	7.7 ± 0.5
VEH + SONL	11	4.3 ± 0.7#	5 ± 1.0#	10	4.3 ± 1.2*	8.2 ± 1.0
VEH + SONR	9	6.5 ± 0.8	3.5 ± 0.4#*	11	7.6 ± 0.8	5.8 ± 1.3
VEH + SONB	8	4.7 ± 0.8	4.8 ± 1.2	8	5.2 ± 0.7	4.7 ± 1.2

ANOVA test, followed by Tukey’s test

*p < 0.05 vs. contralateral ovary in same group

#*p* < 0.05 vs. VEH group

When analyzing histological sections from adult female ovaries treated with VEH at birth, and subjected to bilateral SON section, we observed the presence of corpora lutea and some growing follicles (Fig. 1a). However, ovaries from adult animals treated with TP at birth and subjected to bilateral SON section presented follicular cysts and absence of corpora lutea (Fig. 1a).

In histological sections from adult female ovaries treated with VEH at birth, and subjected to unilateral SON section, we observed the presence of corpora lutea and some growing follicles (Fig. 1b). However, ovaries from adult animals treated with TP at birth and subjected to unilateral SON section presented follicular cysts and absence of corpora lutea, both in denervated ovaries and ovaries with intact innervation (Fig. 1b).

**Fig. 3:**
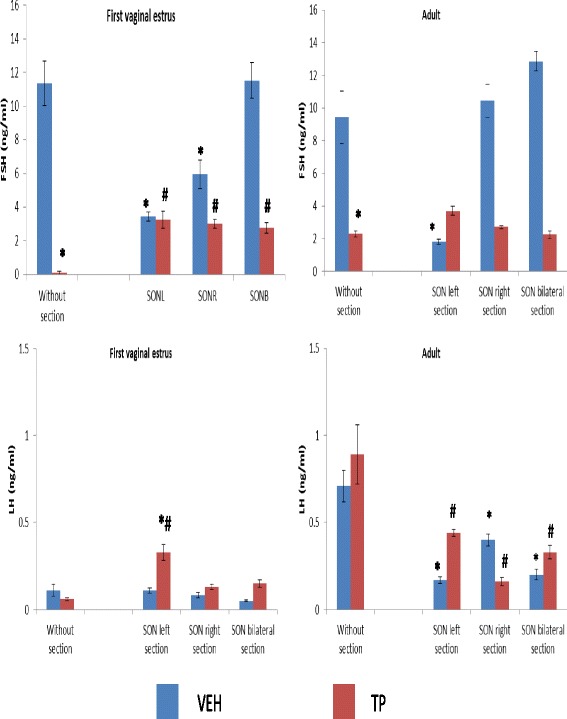
Mean ± s.e.m. FSH and LH serum levels in rat females injected with vehicle (VEH) or testosterone propionate (TP) at birth, and subjected to left superior ovarian nerve section (SONL), right superior ovarian nerve section (SONR), or bilateral superior ovarian nerve section (SONB) at 24 days of age and sacrificed on the first vaginal estrus or in adult age. *p < 0.05 vs. VEH without section, #*p* < 0.05 vs. TP without section. ANOVA followed by Tukey’s test

FSH serum levels in the treatment with TP were lower than in the VEH group, both without SON sections, and both in individuals that were sacrificed on the first vaginal estrus and of adult age (Fig. 3). In rats that were sacrificed on the first vaginal estrus, individuals from the VEH group who had unilateral SON sections showed lower FSH serum levels than individuals without a SON section (Fig. 3). In animals treated with TP at birth, unilateral or bilateral SON sections resulted in higher FSH levels than in animals without the section (Fig. 3). In adult age, left SON sections in VEH-treated animals resulted in lower FSH serum levels than the VEH group without the section (Fig. 3).

Left SON sections in rats treated with TP at birth and sacrificed on the first vaginal estrus resulted in increased LH serum levels compared to the group of animals treated with VEH, or TP without the SON section (Fig. 3). When we analyzed gonadotropin concentrations in adult age, was observed that left SON sections in animals treated with VEH at birth resulted in reduced FSH serum levels compared to the VEH group without the section (Fig. 3). Unilateral or bilateral SON sections in the VEH group resulted in lower LH serum levels than without the section. Unilateral or bilateral SON sections in rats treated with TP at birth showed decreased LH serum levels compared to animals without the section (Fig. 3).

**Fig. 4:**
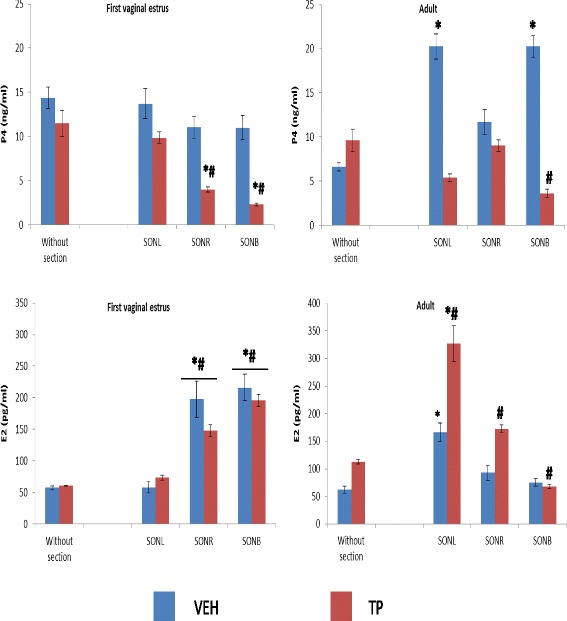
Mean ± s.e.m. progesterone (P4) and estradiol (E2) serum levels in rat females injected with vehicle (VEH) or testosterone propionate (TP) at birth, and subjected to left superior ovarian nerve section (SONL), right superior ovarian nerve section (SONR), or bilateral superior ovarian nerve section (SONB) at 24 days of age and sacrificed on the first vaginal estrus or in adult age. *p < 0.05 vs. VEH without section group. #*p* < 0.05 vs. TP without section group. ANOVA followed by Tukey’s test

A significant decrease in P4 serum levels in TP treated animals subjected to right or bilateral SON sections sacrificed on the first vaginal estrus was observed compared to the VEH or TP groups without sections (Fig. 4). When we analyzed ovarian hormone concentrations in adult age, we observed that the VEH treatment together with left or bilateral SON sections resulted in increased P4 levels compared to the VEH group without sections (Fig. 4). In adult age, animals from the TP group and with bilateral SON sections showed lower serum levels of P4 than animals without sections (Fig. 4).

E2 serum levels in animals sacrificed on the first vaginal estrus treated with VEH or TP at birth, and with right and bilateral SON sections, were higher than in the groups without sections (Fig. 4). In adult age, animals treated with VEH at birth and with left SON sections showed higher E2 serum levels than animals without sections (Fig. 4). In animals treated with TP at birth and with bilateral SON sections, we observed a decrease in E2 serum levels compared to animals without sections (Fig. 4). Finally, animals treated with TP and with unilateral SON sections showed an increase in E2 serum levels compared to animals without sections (Fig. 4).

## Discussion

Our results show that androgenization at birth causes the development of ovarian cysts, reduced FSH concentrations, varying concentrations of steroid hormones, and anovulation, all of which characterize polycystic ovary syndrome. With our experimental model, sympathetic denervation was unable to restore ovulation or hormone secretion.

In the present study, we observed that androgenization using TP at birth induces the development of multiple follicular cysts characterized by a large follicular antrum, hyperthecosis, reduced number of granulosa cells, and, in some cases, ovocytes separated from the cumulus oophorus, absence of corpora lutea, and a few developing follicles, which consequently result in the lack of ovulation. Other researchers have reported similar effects on follicular development by androgens [38–43]. Recently, Anesetti and Chávez-Genaro [7] showed how neonatal administration of testosterone results in the formation of cystic structures characterized by a large antrum, the presence of health ovocytes, and a thick layer of granulosa cells undergoing mitosis. Also, neonatal dihydrotestosterone (DHT) treatment has been observed to lead to the coexistence of lutein-appearing cysts and corpora lutea, and the use of estrogens, such as EV, to induce PCOS, produces follicular ovocyte-lacking cysts, tiny follicles, and anovulation [3, 44, 45]. All of these data provide evidence that supports the idea that ovarian cyst formation depends on the steroid hormone utilized to create the PCOS model, as other authors have also proposed [29, 46–48].

Androgen-induced PCOS has also been found to alter ovarian hormone secretion in a manner dependent on the age of drug administration [6, 7, 49, 50]. In infantile rats, DHT injection results in reduced P4 concentration in adult age [50], while prenatal exposure to T or DHT increases the concentration of the same hormone [6]. Prenatal exposure to T or DHT also increases estradiol concentrations observed in adult rats [6, 51]. Ongaro and collaborators [52] showed that the TP treatment results in an impaired ovary steroidogenic function with a reduced enzyme gene expression of 17OHP4 [52]. This is possibly caused by a drastic reduction in LH and FSH contents at prepubertal age [53]. Furthermore, they showed that in isolated ovarian granulosa cells, the stimulation with hCG results in a hypersecretion of E2 levels in TP rats, in comparison to the control group. Moreover, in basal conditions, the E2 levels were similar in both TP and control groups. The authors found more primary and less antral follicles in the ovaries of juvenile rats (TP-treated), which indicates failure in follicular maturation and, possibly, steroidogenic alterations. This could be explained by the overexpression of the LH receptor related to neonatal androgenization [52].

The results of the estradiol secretion reported in the present study are in accordance with these findings. These results show that the ovarian response to androgens varies according to the animal’s age. This could be due to the amount of gonadal innervations [54], since the participation of the extrinsic innervation of the ovary in the regulation of ovary hormone secretions has already been established by our work group and also by other research groups [31–36, 54].

Classical research has shown that TP administration at different postnatal development stages in rats produces ovarian cysts and ovulation blocking. Consequently, some authors have proposed the hypothesis that PCOS development is caused by androgenic action on gonadotropin-releasing hormone (GnRH) and LH secretion [55, 56]. More recently, it has been postulated that androgens exert an action on hypothalamic centers reprogramming several tissues, including the ovary [5].

Spinedi et al. [53] showed that in the neonatal female rat, the treatment with TP decreases LH-FSH anterior pituitary content and plasma levels, established at 15 days of age. A decreased pattern of pulsatile LH-FSH secretion in plasma is seen during adulthood. The authors suggest that this response can be attributed, at least in part, to a decreased anterior pituitary response to LHRH stimulation, and do not exclude a modification of median eminence LHRH secretion into the portal blood.

Therefore, in the present study, we also analyzed FSH and LH secretion changes at pubertal and adult age in TP-treated animals at birth. Our results show that TP administration at birth modifies the gonadotropin secretion pattern: the first vaginal estrus presented a decrease in FSH serum levels that remained until adult age, whereas LH secretion was found to be normal. Previous research has shown that androgens affect FSH isoforms during the female rat’s newborn stage. As a result, during the adult age, the hypophysis presents a higher proportion of acid FSH isoforms (comparable to those in adult males), so it has been suggested that these isoforms could be involved in the development of follicular cysts in androgenized animals [57]. Androgen exposure has also been shown to induce increased androgen receptor activity in the hypothalamus, thus modifying the gonadotropin secretion pattern, altering follicular growth, blocking ovulation, and ultimately resulting in PCOS development [58]. Our data support the hypothesis that states that early exposure to androgens during individual growth results in the establishment of PCOS [6, 17, 30, 51, 59, 60].

It has been recently suggested that high ovarian sympathetic innervation activity could be an additional explanation for the etiology of the syndrome [2, 30, 48]. Thus, when the ovary sympathetic tone is reduced by bilateral SON section, ovarian morphology is recuperated, cysts disappear, and corpora lutea and growing follicles are observed [29, 48]. In the present investigation, the elimination of sympathetic information by unilateral or bilateral SON section at 24 days of age in rats androgenized at birth did not result in the recovery of the ovarian morphology or of ovulation. It has been shown in other studies that the ovarian noradrenaline content decreases as a response to SON section in prepubertal animals [30, 61]. Our results support the idea that the neonatal period in rats (1–5 days after birth) is the critical window [62, 63] of steroid hormone action on central or peripheral structures producing ovarian function alterations and, in this particular experimental model, the reduced ovary sympathetic tone does not enable the reestablishment of ovary functions.

In prepubertal rats with PCOS, induced with EV, eliminating the main catecholaminergic source by SON bilateral section decreases P4 concentrations [48], and the opposite effect (increased concentrations) is observed when the syndrome is induced in adult age [29]. In previous studies, we showed that the unilateral SON section in EV-treated infantile rats decreases P4 and E2 concentrations [30]. In the present study, animals androgenized at birth and subjected to right or bilateral SON section result in decreased P4 concentrations at first vaginal estrus, with increased E2 concentrations. This response varies when the animal is sacrificed at adult age. In view of these results, it is possible to suggest that in androgenized females, ovary steroidogenesis modulation by the SON depends on the age at which effects are evaluated.

It has been suggested that neural information carried by the SON does not participate in gonadotropin secretion regulation, since, in rats at 24 days of age, SON bilateral section does not modify FSH and LH serum levels evaluated at 31 days of age [33]. Nevertheless, other studies suggest that, in 4 day old rats, the SON participates by stimulating FSH secretion [64]. Our study revealed that in neonatally TP-androgenized rats, the SON modulates LH secretion by inhibition at the time of the first vaginal estrus, while the opposite effect appears to be occurring in adult age. Testosterone propionate treatment decreases FSH serum levels by the first vaginal estrus and also in adult age. Therefore, since unilateral or bilateral SON section increases FSH secretion at the time of the first estrus, we conclude that, at this age, the SON modulates inhibitory FSH secretion, and that the pattern is lost in adult age in animals with PCOS. In rats with VE-induced PCOS, SON bilateral section or electroacupuncture can restore ovarian functions [29, 48, 55]. However, if unilateral section is performed, the denervated ovary does not restore ovulation, but the innervated ovary does [30].

A recent study using global and cell-specific ARKO mouse models with a PCOS mouse model, has shown hyperandrogenism acting directly via the AR, and has provided evidence indicating that the development of PCOS traits does not require AR signaling in granulosa cells, but rather that anovulation, impaired antral follicle, and metabolic disturbance are dependent on a functional neural AR signaling network [28]. Thus, considering that SON section did not restore ovulation in the present study, it is possible to suggest that there are other neural pathways available to androgenized animals which participate in ovarian function regulation. In PCOS induced by EV injection, we showed that unilateral or bilateral section of the vagus nerve restored spontaneous ovulation [65]. Therefore, we presume that the unilateral or bilateral section of the vagus nerve could restore ovulation in PCOS rats induced by TP injection. This issue will be addressed in a future study.

## Conclusions

In conclusion, our results suggest that exposure to testosterone propionate at birth results in the development of PCOS, and, in this model, the syndrome is not due to noradrenergic innervation hyperactivity. This study contributes to a body of evidence of mechanisms of androgens in the development of PCOS. Clarification of the role of androgens in female reproduction is necessary for development of treatments for PCOS.

## Abbreviations

AR: Androgenic receptors
DHT: Dihydrotestosterone
E2: Estradiol
EV: Estradiol valerate
FSH: Follicle stimulating hormone
FVE: First vaginal estrus
GnRH: Gonadotropin-releasing hormone
LH: Luteinizing hormone
NGF: Neural growth factor
P4: Progesterone
PCOS: Polycystic ovary syndrome
SON: Superior ovarian nerve
SONB: Superior ovarian nerve bilateral
SONL: Superior ovarian nerve left
SONR: Superior ovarian nerve right
TP: Testosterone propionate
VEH: Vehicle
